# Assessing the accuracy of thyroid nodule risk-stratification tools in Hispanic and non-Hispanic patients

**DOI:** 10.1210/jendso/bvag078

**Published:** 2026-04-01

**Authors:** Carine Tamamian, Clifford Jiajun He, Charles Choe, Theresa Guo

**Affiliations:** School of Medicine, University of California, San Diego, La Jolla, CA 92037, USA; School of Medicine, University of California, San Diego, La Jolla, CA 92037, USA; Department of Endocrinology, University of California, San Diego, La Jolla, CA 92037, USA; Department of Otolaryngology, University of California, San Diego, La Jolla, CA 92037, USA; Hanna and Mark Gleiberman Head and Neck Cancer Center, Moores Cancer Center, University of California, San Diego, La Jolla, CA 92037, USA

**Keywords:** thyroid nodules, TI-RADS, Bethesda, fine-needle aspiration, molecular testing

## Abstract

**Context:**

Ultrasound risk categories (thyroid imaging, reporting, and data systems, TI-RADS), fine-needle aspiration cytology (Bethesda), and molecular classifiers guide thyroid nodule management. However, these tools may perform differently across populations due to differences in thyroid cancer characteristics among Hispanic patients.

**Objective:**

In this study, we compared the predictive performance of these tools in Hispanic vs non-Hispanic patients using surgical pathology as the gold standard.

**Methods:**

We performed a retrospective study of adult patients (≥18 years) evaluated for thyroid nodules from January 2023 to June 2024 at a tertiary academic medical center. Nodules were stratified using TI-RADS, Bethesda, and molecular testing. Malignancy was confirmed by final surgical pathology, and positive predictive value was assessed within diagnostic strata among resected nodules.

**Results:**

A total of 308 patients (421 nodules) were included in the analytic cohort, with 29.2% being Hispanic. Mean age was 54.3 ± 15.4 years in Hispanic patients and 58.8 ± 16.1 years in non-Hispanic patients (*P* = .02). TI-RADS and Bethesda distributions were similar by ethnicity. Among TI-RADS4 nodules undergoing surgery, malignancy risk was higher in Hispanic patients (32.7% vs 12.0%; odds ratio [OR] 3.57, 95% CI 1.63-7.78; *P* = .001). Among Bethesda III/IV nodules with suspicious molecular results, malignancy was higher in Hispanic patients (78.6% vs 34.8%; adjusted OR 8.72, 95% CI 1.35-96.0; *P* = .022). Overall, Hispanic patients had higher odds of malignancy (OR 2.14, 95% CI 1.26-3.64; *P* = .0047).

**Conclusion:**

Despite similar use of diagnostic tools, Hispanic patients had higher malignancy rates overall and within select diagnostic strata. These findings suggest variation in risk estimate performance across populations and support multicenter validation in diverse cohorts.

Thyroid nodules are becoming increasingly identified through routine imaging [1]. Current clinical practice relies heavily on structured, sequential risk-stratification systems, consisting of ultrasound-based thyroid imaging, reporting, and data systems (TI-RADS); fine-needle aspiration (FNA) with Bethesda cytologic grading; and molecular classifiers to guide decisions regarding surgical biopsy [2]. Both European and American College of Radiology (ACR) TI-RADS frameworks demonstrate adequate overall calibration between assigned TI-RADS categories and observed malignancy rates, as well as substantially reduce unnecessary FNAs [3, 4].

Although these tools and frameworks have demonstrated strong overall performance [2], validation studies were conducted in referral-center populations that do not reflect the full ethnic diversity of patients seen in everyday practice. The detailed racial and ethnic composition of these cohorts has not been consistently reported in these validation studies [3-6], limiting assessment of whether published calibration estimates generalize across diverse patient populations. Disparities in thyroid cancer outcomes across ethnic groups have been documented [3], further raising questions regarding whether diagnostic performance metrics derived from homogeneous cohorts are generalizable to diverse patient populations. For instance, 1 retrospective study found that the positive predictive value (PPV) of the Bethesda system varied across racial and ethnic groups, with African American patients showing the lowest PPV compared with Hispanic and White patients, highlighting that diagnostic accuracy may differ by population [7].

In a large public hospital cohort supplemented with population-based data, Moo-Young et al showed that Hispanic patients with differentiated thyroid cancer (mostly papillary) had significantly higher rates of lymph node involvement and metastatic disease at diagnosis compared with non-Hispanic White patients [8]. Similarly, a study of the California Cancer Registry demonstrated that Hispanic patients present with a higher rate of advanced thyroid cancer at diagnosis, as well as an increased mortality risk [9]. These patterns highlight a need to evaluate whether existing risk-stratification tools perform accurately in Hispanic populations. Because these systems assign malignancy probability using population-derived estimates, differences in baseline disease prevalence or clinical presentation may alter their predictive performance in specific populations.

Given these previously reported disparities, this study sought to determine whether widely used thyroid nodule risk-stratification systems perform equitably among Hispanic and non-Hispanic groups. In this study, we assess whether TI-RADS, Bethesda cytology, and molecular classifiers provide comparable malignancy risk prediction in Hispanic and non-Hispanic patients at a tertiary academic center. Importantly, our objective is not to treat ethnicity as an isolated exposure, but to test whether commonly used tools show differences in real-world performance and calibration across patient populations, which could influence clinical interpretation and downstream management. We hypothesized that the performance of commonly used thyroid nodule diagnostic tools would differ between Hispanic and non-Hispanic groups.

## Materials and methods

### Study population

**Figure 1:**
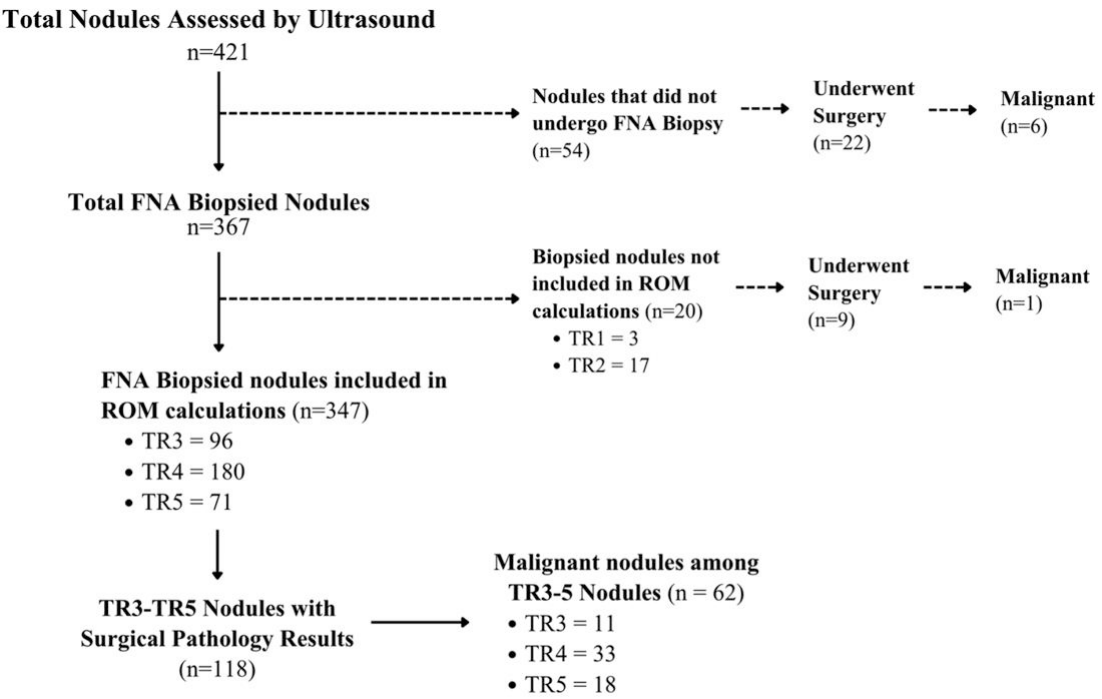
Flow diagram illustrating thyroid nodule inclusion and exclusion for ROM analyses. The diagram depicts the total number of nodules assessed by ultrasound, nodules undergoing FNA, exclusions from ROM calculations (including TI-RADS TR1-2 nodules), and the subset of TR3 to TR5 nodules with available surgical pathology used to calculate ROM.

We conducted a retrospective chart review of adult patients presenting with thyroid nodules at a tertiary medical center under Institutional Review Board (IRB) protocol #810404. Adult patients (≥18 years) evaluated for thyroid nodules between January 2023 and June 2024 were screened for inclusion. Patients were eligible if they had at least 1 thyroid nodule that underwent diagnostic evaluation with ultrasound (Fig. 1). Nodules were included in the analytic cohort if they had pathologic evaluation, defined as either Bethesda cytology from FNA or final surgical pathology available for the index nodule.

Demographic variables were extracted from the electronic medical record at the time of the nodule evaluation. These included age, sex, and ethnicity (Hispanic vs non-Hispanic). Additional clinical variables collected included nodule size. All demographic variables were analyzed at the patient level, whereas nodule-specific data (Nodule size, TI-RADS, Bethesda, molecular testing, and surgical pathology) were analyzed at the nodule level.

### TI-RADS scoring

Patients were recommended FNA biopsy if their nodules met the following criteria: TR3 and ≥2.5 cm, TR4 and ≥1.5 cm, or TR5 and ≥1.0 cm [3].

The majority of ultrasounds were performed at our institution; for studies performed outside the institution, ultrasound reports were available and used for evaluation. All nodules were classified according to ACR TI-RADS criteria based on the ultrasound reports. Some patients decide to not undergo a biopsy despite meeting criteria and undergo direct surgery. Other patients chose to observe their nodules with no further intervention despite qualifying for biopsy. These decisions were influenced by individual patient preferences, risk perceptions, prior experiences, and discussions with their physicians.

### FNA Bethesda scoring

Thyroid nodule FNA cytology results were reported using the Bethesda System for Reporting Thyroid Cytopathology. The Bethesda categories range from I (nondiagnostic/unsatisfactory) and II (benign) to III and IV (indeterminate), V (suspicious for malignancy), and VI (malignant) [10]. Management decisions were guided by these categories. Benign (Bethesda II) nodules were typically routinely observed, while indeterminate or suspicious nodules (Bethesda III-V) were advised to undergo additional molecular testing [11]. The results of molecular testing were incorporated into subsequent management decisions such as observation or surgery.

### Molecular testing

Indeterminate and suspicious nodules (Bethesda III-V) were eligible for molecular testing at the discretion of the treating clinicians. Two platforms were used at our institution: the Afirma Genomic Sequencing Classifier and ThyroSeq v3. Afirma Genomic Sequencing Classifier classifies nodules as benign or suspicious using RNA expression profiling via next-generation sequencing [12], while ThyroSeq v3 assesses DNA and RNA for common thyroid-related mutations and fusions [13]. ThyroSeq results were interpreted using both the mutation profile and the genomic classifier output. A ThyroSeq result was categorized as suspicious if the report was explicitly labeled as “positive,” and if either a pathogenic/likely pathogenic mutation was identified (eg, BRAF V600E, HRAS p.Q61, GLIS1/PAX8, RET/PTC1) or if the genomic classifier predicted a malignancy risk ≥50% [13]. Results showing no pathogenic alteration and a genomic classifier risk <50%, or explicitly reported as “negative,” were categorized as benign in our cohort.

### Surgical management

A subset of patients underwent surgical intervention, either lobectomy or total thyroidectomy, which was commonly determined by suspicious FNA and molecular testing results or symptomatic nodules. Surgical pathology, considered the gold standard, provided definitive histopathological diagnoses [14]. Each resected nodule was classified as benign or malignant postsurgery.

Final surgical pathology was used to calculate overall malignancy and the risk of malignancy (ROM), allowing for the assessment of preoperative FNA and molecular testing accuracy. Overall malignancy is defined as the proportion of surgically malignant nodules among all nodules evaluated by ultrasound. Risk of malignancy was defined as the proportion of malignant nodules within each TI-RADS, Bethesda, and molecular testing category among nodules that proceeded to surgery with available surgical pathology results. The analytic dataset included only the index thyroid nodule that prompted diagnostic evaluation. In some cases, additional nodules were identified on surgical pathology, but these were not captured in the dataset and were not included in the analysis. Only nodules that underwent FNA biopsy and surgical resection were included in ROM calculations. Accordingly, ROM values should be interpreted as conditional PPV among resected nodules rather than true population prevalence.

### Statistical analysis

The primary outcomes of interest were differences in diagnostic performance and management of thyroid nodules by ethnicity, with a focus on the PPV of commonly used diagnostic tools. Positive predictive value was defined as the proportion of nodules with a positive diagnostic test result that were confirmed malignant on final surgical pathology. For ultrasound- and cytology-based diagnostic tools, PPV was operationalized as the ROM within each diagnostic category among nodules that underwent FNA and subsequent surgery. Analyses of cytology-based diagnostic performance focused primarily on Bethesda III and IV nodules, as these nodules carry intermediate risk and require further evaluation through molecular testing and surgery [2]. Risk of malignancy was calculated for TI-RADS categories TR3 to TR5 and for Bethesda cytology categories III to VI. For molecular testing analyses, PPV/ROM was defined as the proportion of nodules with suspicious or positive molecular test results that were malignant on final surgical pathology.

Categorical variables were summarized as counts and percentages and compared between Hispanic and non-Hispanic patients using χ^2^ tests. Continuous variables were summarized as means with SDs and compared using independent-sample *t*-tests. Logistic regression models were used to estimate odds ratios (ORs) and 95% CIs for comparisons between Hispanic and non-Hispanic patients across diagnostic and management outcomes. Unadjusted logistic regression was used for the analyses of ROM/PPV within TI-RADS and Bethesda diagnostic categories, as these analyses were descriptive in nature. Multivariable logistic regression models, adjusted for age, sex, and ultrasound nodule size, were used to evaluate biopsy completion and molecular testing–related outcomes when sample size permitted. For adjusted biopsy completion analyses, generalized linear mixed models with a random intercept for patient ID were used to account for clustering of multiple nodules within patients. For analyses with small cell counts, unadjusted models were used. All statistical analyses were performed using JMP Pro version 18, and statistical significance was defined as a 2-sided *P*-value of <.05. Analyses were performed at the nodule level, as some patients contributed >1 thyroid nodule. As a result, nodule-level observations may not be fully independent, and effect estimates should be interpreted with this consideration in mind.

## Results

### Patient characteristics

A total of 308 patients were included in the analysis, with 90 (29.2%) identifying as Hispanic and 218 (70.8%) as non-Hispanic. Among Hispanic patients, 74 (82.2%) were female and 16 (17.8%) were male. Among non-Hispanic patients, 175 (80.3%) were female and 43 (19.7%) were male (Table 1). Among non-Hispanic patients, racial distribution was as follows: 139 (63.8%) White, 37 (17.0%) Asian, 12 (5.5%) Black, 3 (1.4%) American Indian, 24 (11.0%) other races, and 3 (1.4%) unknown. The Hispanic cohort was defined based on self-identified ethnicity in the electronic medical record and was largely categorized as “other” or “mixed race,” limiting more detailed characterization of subgroup diversity and potentially affecting generalizability.

**Table 1:** Patient count and demographic summary for adult patients being evaluated for thyroid nodules

Variable	Hispanic *n* (%)	Non-Hispanic *n* (%)	*P*-value
Total	90 (29.2)	218 (70.8)	—
Sex			.69*^a^*
Female	74 (82.2)	175 (80.3)	
Male	16 (17.8)	43 (19.7)	
Age, years ± SD	54.3 ± 15.4	58.8 ± 16.1	**.02** *^b^*
18-29	7 (7.8)	8 (3.7)	
30-49	25 (27.8)	58 (26.6)	
50-64	33 (36.7)	55 (25.2)	
65 and older	25 (27.8)	97 (44.5)	
Preferred language			**<.001** *^a^*
English	59 (65.5)	200 (91.7)	
Spanish	30 (33.3)	4 (1.8)	
Other	1 (1.1)	14 (6.4)	
Ultrasound location			**.622**
UCSD	76 (84.4)	179 (82.11)	
Outside institution	14 (15.6)	39 (17.9)	

Significant *P*-values in bold.

Abbreviation: UCSD, University of California, San Diego.

^*a*^ *P*-values for categorical variables were calculated using χ^2^ test.

^*b*^ *P*-values for continuous variables were calculated using Student’s *t*-test.

Hispanic patients with thyroid nodules were more likely to present at a younger age, with the mean age of 54.3 ± 15.4 years compared with 58.8 ± 16.1 years (*P* = .02). Hispanic patients were more likely to present between ages 50 and 64 compared with non-Hispanic patients (36.7% vs 25.2%). The majority of ultrasounds were performed at our institution for both Hispanic and non-Hispanic patients (84.4% vs 82.1%). Studies performed externally were still incorporated into the nodule management decision-making process. Language preference also differed significantly between the groups, where 91.7% of non-Hispanic patients reported English as their preferred language compared with 65.5% of Hispanic patients (*P* < .001). After English, the most common preferred language for Hispanic patients was Spanish (33.3% of patients).

### Nodule and biopsy characteristics

A total of 421 nodules were included in the analysis (Fig. 1). The distribution of TI-RADS categories did not differ by ethnicity, with TR4 being the most common classification in both groups (48.4% vs 45.7%), followed by TR3 (31.3% vs 26.6%) and TR5 (16.4% vs 19.8%). The distribution of Bethesda cytology categories also did not differ by ethnicity. Bethesda II was the most frequent diagnosis in both Hispanic and non-Hispanic patients (50.5% vs 62.5%), followed by Bethesda III and IV (27.2% vs 20.9%) and Bethesda V and VI (18.4% vs 12.9%; Table 2).

**Table 2:** Nodule counts and diagnostic distribution across TI-RADS categories and Bethesda cytology grades by patient ethnicity

Variable	Hispanic, *n* (%)	Non-Hispanic, *n* (%)	*P*-value
TI-RADS	*n* = 128	*n* = 293	.33
TR1-2	5 (3.9)	23 (7.9)	
TR3	40 (31.3)	78 (26.6)	
TR4	62 (48.4)	134 (45.7)	
TR5	21 (16.4)	58 (19.8)	
Bethesda grades	*n* = 103	*n* = 264	.14
Category I	4 (3.9)	10 (3.8)	
Category II	52 (50.5)	165 (62.5)	
Category III	21 (20.4)	49 (18.6)	
Category IV	7 (6.8)	6 (2.3)	
Category V	13 (12.6)	25 (9.5)	
Category VI	6 (5.8)	9 (3.4)	
Overall malignancy	31 (24.2)	38 (12.9)	**.005**

*P*-values for categorical variables calculated using χ^2^ test. The table presents the count and percentage of nodules classified within each thyroid imaging, reporting, and data system (TI-RADS) level (TR1-TR5) and each Bethesda grade (I-VI) in the study cohort. Because some patients had multiple nodules, nodule-level analyses exceed patient counts. Overall malignancy was calculated as the proportion of malignant nodules among all nodules evaluated with ultrasound. Significant statistics in bold.

Hispanic patients had larger thyroid nodules on ultrasound compared with non-Hispanic patients on average (2.77 ± 1.58 vs 2.43 ± 1.37 cm, *P* = .036, Table 3). Although Hispanic patients were more likely to have nodules recommended for biopsy based on TI-RADS criteria, this difference did not reach statistical significance (75.8% vs 67.2%, *P* = .084). Among nodules for which biopsy was recommended, biopsy completion was significantly lower among Hispanic patients compared with non-Hispanic patients (85.6% vs 94.4%, *P* = .022). After adjustment for age, sex, ultrasound nodule size, and clustering of nodules within patients, Hispanic ethnicity was independently associated with lower odds of biopsy completion, both among all nodules (adjusted OR 0.46, 95% CI 0.22-0.93; *P* = .033) and among nodules recommended for biopsy (adjusted OR 0.32, 95% CI 0.12-0.79; *P* = .015; Table 4).

**Table 3:** Nodule evaluation metrics summarizing differences in thyroid nodule evaluation and biopsy completion between Hispanic and non-Hispanic patients

Outcome	Hispanic, *n* (%)	Non-Hispanic, *n* (%)	*P*-value
Nodules recommended for biopsy	97/128 (75.8)	197/293 (67.2)	.084*^a^*
Biopsy completed among recommended nodules only	83/97 (85.6)	186/197 (94.4)	.**022***^a^*
Biopsy completed among all nodules	103/128 (80.5)	264/293 (90.1)	.083*^a^*
Mean nodule size on ultrasound (cm) ± SD	2.77 ± 1.58	2.43 ± 1.37	.**036***^b^*

^a^ *P*-values for categorical variables were calculated using χ^2^ test, significant values in bold.

^*b*^ *P*-values for continuous variables were calculated using Student’s *t*-test, significant values in bold.

**Table 4:** Adjusted odds ratios for biopsy completion by ethnicity

Outcome	Adjusted OR	95% CI	*P*-value
Biopsy completed among all nodules*^a^*	0.46	0.22-0.93	.**033**
Biopsy completed among recommended nodules only	0.32	0.12-0.79	.**015**

Multivariable logistic regression models were adjusted for ultrasound nodule size, age, and sex. Odds ratios (ORs) represent the odds of the outcome among Hispanic patients compared with non-Hispanic patients (reference group). Confidence intervals represent 95% confidence limits for each estimate. Significant *P*-values in bold.

^*a*^Adjusted odds ratios for biopsy completion among all nodules were estimated using a generalized linear mixed model with a random intercept for patient ID to account for clustering of multiple nodules within patients. The model was adjusted for age, sex, and ultrasound nodule size. For analyses restricted to nodules recommended for biopsy, standard multivariable logistic regression was used because mixed-effects models did not converge.

In exploratory analyses, preferred language was examined as a predictor of biopsy completion. While unadjusted analyses suggested lower completion rates among Spanish-speaking patients, this association was not significant after adjustment in a multivariable generalized linear mixed model accounting for clustering by patient (*P* = .21). In contrast, when both language and ethnicity were included in the model, Hispanic ethnicity remained independently associated with lower odds of biopsy completion (OR 0.41, 95% CI 0.19-0.86; *P* = .018), suggesting that the observed disparity was not explained by language alone.

### Increased risk of malignancy in Hispanic patients

Hispanic patients were more than twice as likely to have malignant thyroid nodules compared with non-Hispanic patients (OR = 2.14, 95% CI 1.26-3.64, *P* = .005; Table 2). We next compared tool-stratified ROM (conditional on surgery) to evaluate real-world diagnostic performance across groups (Table 5). When stratified by TI-RADS category, Hispanic patients demonstrated a significantly higher ROM among TR4 nodules compared with non-Hispanic patients (32.7% vs 12.0%; OR 3.57, 95% CI 1.63-7.78; *P* = .001). No statistically significant differences in ROM were observed for TR3 or TR5 nodules. When stratified by Bethesda cytology category, ROM was significantly higher among Hispanic patients across Bethesda III/IV nodules (46.4% vs 23.6%; OR 2.80, 95% CI 1.06-7.38; *P* = .037; Table 5). For Bethesda V/VI nodules, the ROM did not differ significantly between Hispanic and non-Hispanic patients (73.7% vs 58.8%; OR 1.96, 95% CI 0.57-6.69; *P* = .28).

**Table 5:** Risk of malignancy among thyroid nodules that underwent FNA, stratified by TI-RADS category (TR3-TR5), Bethesda cytology (III-VI), and ethnicity

	Hispanic			Non-Hispanic, *n* (%)			OR	95% CI	*P*-value
	Biopsied, *n*	Malignant, *n*	ROM, %	Biopsied, *n*	Malignant, *n*	ROM, %			
Variable									
TR3	27	5	18.5	69	6	8.7	2.39	0.66-8.58	.18
TR4	55	18	32.7	125	15	12.0	3.57	1.63-7.78	.**001**
TR5	18	5	27.8	53	13	24.5	1.18	0.35-3.95	.79
Cytology									
Bethesda III/IV	28	13	46.4%	55	13	23.6%	2.80	1.06-7.38	.**037**
Bethesda V/VI	19	14	73.7%	34	20	58.8%	1.96	0.57-6.69	.28

“Biopsied” refers to nodules that underwent fine-needle aspiration (FNA) and subsequently proceeded to surgery with available surgical pathology. “Malignant” refers to nodules confirmed malignant on final surgical pathology. Risk of malignancy (ROM) represents the proportion of biopsied nodules within each thyroid imaging, reporting, and data system (TI-RADS) category that were malignant. Significant *P*-values in bold.

In secondary analyses stratified by ethnicity, and adjusted for age and sex, TI-RADS was significantly associated with malignancy among non-Hispanic patients (likelihood ratio χ^2^ = 19.13, df = 4, *P* = .0007). In contrast, no statistically significant association between TI-RADS category and malignancy was observed among Hispanic patients after adjustment (likelihood ratio χ^2^ = 2.54, df = 3, *P* = .47).

### Impact of molecular testing

**Figure 2:**
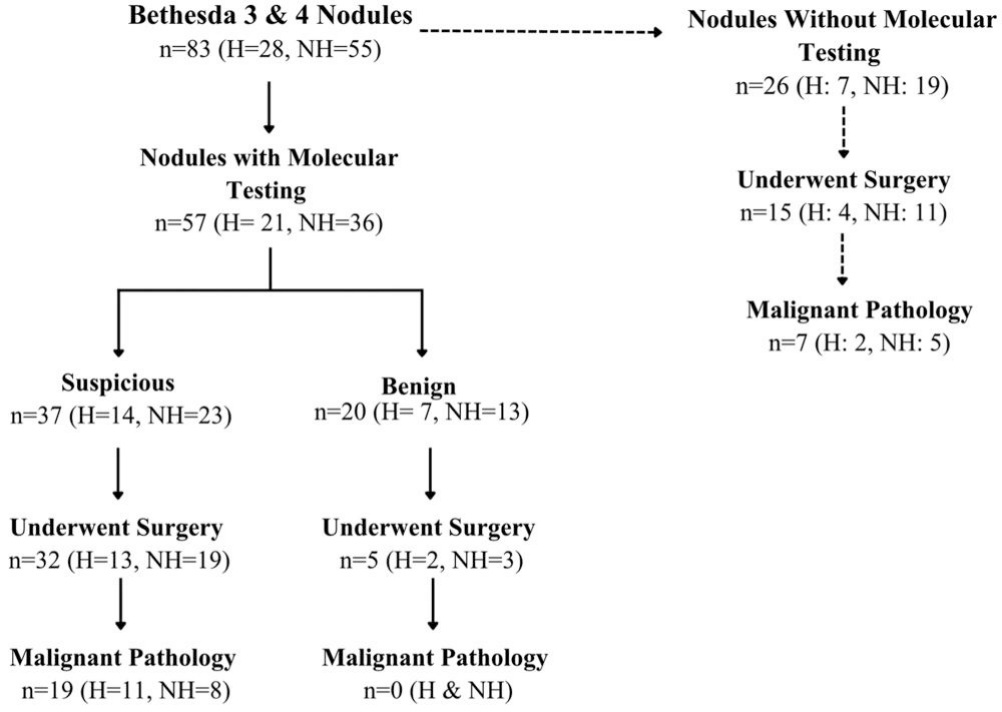
Flow diagram illustrating management pathways for Bethesda III and IV thyroid nodules, stratified by molecular testing status and ethnicity. Nodules are categorized by whether molecular testing was performed, subsequent molecular test results (suspicious vs benign), progression to surgery, and final surgical pathology. Counts are shown for the overall cohort and separately for Hispanic (H) and non-Hispanic (NH) patients.

Among Bethesda III and IV nodules, rates of molecular testing did not differ significantly by ethnicity (75.0% in Hispanic patients vs 65.5% in non-Hispanic patients; *P* = .546; Table 6, Fig. 2). The proportion of nodules classified as suspicious (or positive) on molecular testing was also not significantly different between the 2 groups (66.7% vs 63.9%; *P* = .583).

**Table 6:** Molecular testing use and surgical outcomes among Bethesda III and IV thyroid nodules, stratified by ethnicity

Variable	Hispanic, *n* (%)	Non-Hispanic, *n* (%)	OR	95% CI	*P*-value
Bethesda III and IV	*n* = 28	*n* = 55			
Genetic testing rate	21/28 (75.0)	36/55 (65.5)	1.39*^a^*	0.48-4.26	.546
Suspicious/positive nodules on molecular testing	14/21 (66.7)	23/36 (63.9)	1.42*^a^*	0.41-5.29	.583
Malignancy confirmed at surgery (in nodules suspicious by molecular testing)	11/14 (78.6)	8/23 (34.8)	8.72*^a^*	1.35-96.0	.**022**
Direct surgery without molecular testing*^b^*	4/28 (14.3)	11/55 (20.0)			
Malignancy confirmed at surgery (no molecular testing)	2/4 (50.0)	5/11 (45.5)	0.83*^c^*	0.08-9.16	.88

Values are reported as *n* (%). Odds ratios (ORs) compare Hispanic to non-Hispanic patients. Signifcant *P*-values in bold.

^*a*^Adjusted for age, sex, and ultrasound nodule size.

^*b*^Descriptive rows; no regression performed.

^*c*^Unadjusted logistic regression due to small sample size.

Among nodules with suspicious molecular testing results that subsequently underwent surgery, ROM was significantly higher in Hispanic patients compared with non-Hispanic patients (78.6% vs 34.8%; adjusted OR 8.72, 95% CI 1.35-96.0; *P* = .022). Rates of direct surgery without molecular testing were comparable between the 2 groups, and ROM among these nodules did not differ significantly by ethnicity.

## Discussion

In this retrospective cohort study, we identified important differences in the performance of commonly used thyroid nodule risk-stratification tools between Hispanic and non-Hispanic patients. Although TI-RADS categories, Bethesda cytology classifications, and molecular testing utilization were applied similarly among Hispanic and non-Hispanic patients, important disparities in malignancy risk were observed. Overall malignancy on final surgical pathology was higher among Hispanic patients, indicating that differences in cancer rates persisted in our cohort. Together, these patterns raise concern for population-specific differences in risk stratification, which is often interpreted and calibrated as generalizable across clinical settings. These disparities were most evident among TR4 nodules, indeterminate Bethesda III/IV nodules, and nodules with suspicious molecular testing results. Furthermore, Hispanic patients were younger and presented with larger thyroid nodules compared with non-Hispanic patients. While these differences in presentation may suggest higher baseline malignancy risk among Hispanic patients, or lower screening thresholds in non-Hispanic patients, the observed differences persisted within the same diagnostic categories (such as TI-RADS 4 and Bethesda III/IV), suggesting that patient selection alone does not fully explain our findings.

When examining differences in ultrasound-based risk stratification, Hispanic patients with TR4 nodules demonstrated significantly higher ROM compared with non-Hispanic patients. This difference was observed despite similar distributions of TI-RADS categories between groups, indicating that disparities in malignancy risk were not driven by differential ultrasound classification alone. Consistent with this finding, stratified and adjusted analyses demonstrated that TI-RADS category was significantly associated with malignancy among non-Hispanic patients, whereas no statistically significant association was observed among Hispanic patients after adjustment for age and sex. The ACR TI-RADS guidelines estimate malignancy risk for TR4 nodules at ∼5% to 20% [3]. Multi-institutional validation studies have reported aggregate malignancy rates for TR4 nodules of ∼9.1% [5]. In our cohort, the observed ROM among non-Hispanic patients with TR4 nodules was 12.0%, aligning with both the guideline-predicted range and previously published validation data. In contrast, the observed ROM among Hispanic patients with TR4 nodules was 32.7%, substantially exceeding both the upper bound of the guideline-estimated range (20%) and published validation estimates.

Although overclassification within TR4 has been previously recognized [15], our findings suggest that this alone does not explain the observed disparity. The TR4 assignment was similar across Hispanic and non-Hispanic patients, yet malignancy risk within TR4 differed markedly by ethnicity. This pattern was not random variation but a consistent ethnicity-associated difference within nearly identical diagnostic categories, supporting the possibility of population-level differences in calibration of TI-RADS risk estimates.

When stratified by Bethesda cytology category, ROM was higher for Bethesda III/IV nodules in Hispanic patients compared with non-Hispanic patients. Consistent with our results, prior research has demonstrated that malignancy risk estimates for indeterminate thyroid nodules vary substantially across clinical practice settings. In a multi-institutional study evaluating the Afirma gene expression classifier among Bethesda III and IV nodules, Marti et al found variation in PPV driven by differences in baseline malignancy prevalence, despite similar cytologic classification and test application across institutions [16]. Importantly, Bethesda III and Bethesda IV nodules are typically associated with estimated malignancy risks of ∼6% to 18% and 10% to 40%, respectively [10]; in our cohort, the observed ROM among resected Bethesda III/IV nodules was 46.4% in Hispanic patients (vs 23.6% in non-Hispanic patients). This variability underscores the importance of baseline malignancy prevalence when interpreting malignancy risk within indeterminate cytologic categories. However, due to the relatively small subgroup sample size, these estimates should be interpreted with caution.

Differences in molecular testing performance further supported the observed disparities in diagnostic risk stratification. Despite comparable rates of molecular testing between groups, the risk of malignancy among nodules classified as suspicious on molecular testing was significantly higher in Hispanic patients compared with non-Hispanic patients (78.6% vs 34.8%, *P* = .022). This finding suggests that the PPV of molecular classifiers may vary by underlying population malignancy prevalence or population-specific factors, rather than differences in test utilization alone. Prior meta-analytic data demonstrate substantial variability in the PPV of molecular tests across institutional cohorts, driven by differences in baseline malignancy prevalence, highlighting the importance of population context when interpreting molecular testing results [17]. In a clinical setting that serves a substantial Hispanic patient population, our findings emphasize how population- and ethnicity-specific disease prevalence may influence the real-world performance of molecular diagnostic tools.

Population-based studies further contextualize these findings. Moon et al reported higher rates of advanced thyroid cancer at diagnosis among Native Hawaiian and Pacific Islander patients compared with non-Hispanic White and Asian patients [18]. Moreover, analyses from the California Cancer Registry have shown that Hispanic patients with thyroid cancer are more likely to present with advanced thyroid cancer characteristics and experience worse outcomes compared with non-Hispanic White patients [9]. While these studies focused on disease presentation and outcomes rather than diagnostic performance, they highlight important population-level differences in thyroid cancer that motivate examination of whether commonly used diagnostic and risk-stratification tools perform uniformly across underrepresented groups.

In addition to differences in malignancy risk within diagnostic strata, Hispanic patients in our cohort were statistically less likely to complete recommended thyroid nodule biopsy after adjustment for age, sex, and nodule size. Importantly, biopsy completion rates remained high in both groups, supporting the validity of downstream analyses. This indicates that modest disparities in FNA biopsy follow-up may coexist with differences in diagnostic risk estimation, potentially compounding delays in biopsy and surgery. While this study was not designed to identify causal mechanisms, factors such as language barriers, access to care, scheduling challenges, or patient-provider communication may warrant further investigation. Notably, lower biopsy completion among patients with higher observed malignancy risk highlights the need to improve diagnostic follow-up in underserved groups.

Our findings have important clinical implications for the evaluation of thyroid nodules in ethnically diverse populations. Despite similar distributions of TI-RADS categories, Bethesda cytology classifications, and molecular testing utilization, Hispanic patients demonstrated higher observed malignancy risk within specific diagnostic strata. These results suggest that malignancy risk estimates embedded within commonly used thyroid nodule risk-stratification tools may not perform uniformly across diverse patient populations. In clinical practice, our results highlight the importance of integrating patient-level clinical context, such as ethnicity, alongside the standard risk-stratification tools.

### Strengths and limitations

This study has several strengths. We analyzed a well-characterized cohort from a tertiary academic cancer center, with detailed integration of ultrasound findings, cytologic classification, molecular testing results, and final surgical pathology, allowing for robust assessment of diagnostic performance across multiple stages of thyroid nodule evaluation. Additionally, biopsy completion rates remained high overall, supporting the validity of our analysis.

Several limitations should be acknowledged. First, the retrospective and single-center design may limit generalizability to other practice settings with different patient populations. Second, ROM/PPV estimates are conditional on surgical verification (verification/workup bias): nodules proceeding to surgery are not a random subset of all evaluated nodules, and differences in referral or follow-through could influence observed PPV/ROM. Third, while we identified differences in diagnostic performance by ethnicity, this study was not designed to determine underlying causal mechanisms, including biological, social, or structural contributors.

Despite these limitations, the consistency of observed patterns across ultrasound-based classification, cytology, and molecular testing strengthens the validity of our findings.

### Conclusion

In this study, Hispanic patients demonstrated significantly higher overall malignancy rates than non-Hispanic patients despite similar TI-RADS and Bethesda category distributions. Disparities in malignancy risk emerged within specific diagnostic steps, particularly in TR4 nodules and in nodules with suspicious molecular testing results, suggesting that population-level differences may influence the real-world predictive performance of commonly used thyroid nodule risk-stratification tools. Future studies should assess diagnostic tool performance in larger, multicenter cohorts and examine how population-level factors influence thyroid nodule evaluation, with the goal of refining risk-stratification strategies and promoting equitable care.

## Data Availability

Some or all datasets generated during and/or analyzed during the current study are not publicly available but are available from the corresponding author on reasonable request.

## Abbreviations

ACR: American College of Radiology
FNA: fine-needle aspiration
OR: odds ratio
PPV: positive predictive value
ROM: risk of malignancy
TI-RADS: thyroid imaging, reporting, and data systems
